# Lyme Disease among Patients at an Ambulatory Unit in a Highly Endemic Country: Lithuania

**DOI:** 10.3390/medicina57020184

**Published:** 2021-02-21

**Authors:** Agnė Petrulionienė, Daiva Radzišauskienė, Algimantas Paulauskas, Algirdas Venalis

**Affiliations:** 1 Clinic of Rheumatology, Orthopaedics Traumatology and Reconstructive Surgery, Institute of Clinical Medicine, Faculty of Medicine, Vilnius University, 01513 Vilnius, Lithuania; 2 Clinic of Infectious Diseases and Dermatovenerology, Institute of Clinical Medicine, Faculty of Medicine, Vilnius University, 01513 Vilnius, Lithuania; daiva730jvg@gmail.com; 3 Department of Biology, Vytautas Magnus University, 44248 Kaunas, Lithuania; algimantas.paulauskas@vdu.lt; 4 Faculty of Medicine, Vilnius University, 01513 Vilnius, Lithuania; algirdas.venalis@imcentras.lt

**Keywords:** erythema migrans, Lithuania, Lyme arthritis, Lyme disease, tick-borne diseases

## Abstract

*Background and objectives:* Lyme disease is the most common tick-borne infectious disease in Europe, caused by the spirocheta bacteria of *Borrelia burgdorferi.* Several genospecies of *B. burgdorferi* are pathogenic to humans. *B. burgdorferi* sensu stricto, which is prevalent in North America, causes reactive arthritis, whereas *B. garinii* and *B. afzelii,* common in Europe, can affect the skin, heart, or nervous system; it has been shown that the clinical symptoms of the disease may be very different. The objective of this study was to identify the baseline characteristics of Lyme disease and to elucidate the frequency of different Lyme disease syndromes in Lithuania. *Materials and Methods:* Patients who were diagnosed with Lyme disease during an ambulatory visit to the Center of Infectious Diseases, Vilnius University Santaros clinics, from 2014 to 2016, were enrolled in this study. A retrospective material analysis was conducted. *Results:* In total, 1005 patients were enrolled with the following prevalence of clinical syndromes: erythema migrans (EM), 945 (94.02%); Lyme arthritis, 32 (3.18%); neuroborreliosis, 23 (2.28%); Lyme carditis, 4 (0.39%); and acrodermatitis, 1 (0.09%). Erythema migrans was dominant among middle-aged women, with a rash appearing mainly on the lower extremities. Lyme arthritis mainly manifested among middle-aged women as an oligoarthritis, mostly affecting the knee joint. Neuroborreliosis was seen more often in middle-aged women than men and the main symptom was nervus facialis neuropathy. Lyme carditis, manifested as an atrioventricular block, with a male/female ratio of 3:1, and the median age was 51. Acrodermatitis was diagnosed in a 61-year-old woman, as a painful, red rash on the hand. *Conclusions:* According to the prevalence of *B. garinii* and *B. afzelii* in Europe, previously it was thought that Lyme disease presented as erythema migrans, and less frequently as neuroborreliosis; however, this study revealed that other syndromes may also be seen. In addition, we revealed that the longer it takes for erythema migrans to appear, the greater the likelihood of Lyme arthritis developing.

## 1. Introduction

Lyme disease is the most common tick-borne infectious disease in Europe caused by spirocheta bacteria of *Borrelia burgdorferi. B. Burgdorferi* is subdivided into multiple *Borrelia* species, of which three can cause human infection, i.e., *B. burgdorferi* sensu stricto (ss), *Borrelia afzelii,* and *Borrelia garinii* [1]. *B. burgdorferi* ss is prevalent in North America, while *B. garinii* and *B. afzelii* are more prevalent in Europe. Worldwide, varied prevalence of *B. burgdorferi* subtypes has led to different disease clinical presentations according to geographical areas. *B. burgdorferi* ss is associated with Lyme arthritis, while *B. garinii* leads to neurological disorders (e.g., neuroborreliosis), and *B. afzelii* is associated with skin disorders (e.g., acrodermatitis chronica atrophicans). The clinical manifestations of Lyme disease range from a localized infection, for example, erythema migrans (EM) to a disseminated neurological or rheumatological infection, cardiac involvement, or even chronic disease [2]. Other non-specific symptoms may accompany Lyme disease including flu-like symptoms such as arthralgia, myalgia, fever, headache, and general weakness [3].

Laboratory diagnostic tests for Lyme disease are limited. Blood analysis for Lyme immunoglobulins using enzyme-linked immunosorbent assays (ELISA) or Western blot are usually performed; however, the results can be false positive or false negative. In endemic areas, about 30% of people are positive for Lyme immunoglobulins and, therefore, it is difficult to interpret the results. Therefore, it is important to know the clinical disease characteristics to avoid a misdiagnosis that can lead to late disseminated or even persistent and progressive disease and to avoid overdiagnosis that can lead to overuse of antibiotics and may cause antibiotic resistance. While there is a lack of definitive testing methods to confirm a diagnosis of Lyme disease, knowing the clinical course of the disease is important.

Lyme disease is common in Lithuania. The average crude incidence rate of Lyme borreliosis (LB), in Lithuania, was 85.4, from 2014 to 2016 [4]. According to recent climate changes, when winters become milder, ticks are active for a longer period of time and, therefore, the risk of a tick bite and being infected with *B. burgdorferi* is even higher.

The objective of this work is to clarify the clinical symptoms of Lyme disease, in Lithuania, to identify the most common Lyme disease syndromes, and to review the use of antibiotics.

## 2. Materials and Methods

All adult patients, who were diagnosed with Lyme disease (A69.2) during an ambulatory visit to the Center of Infectious Diseases, Vilnius University Santaros clinics from 2014 to 2016, were enrolled in this study. This hospital is the reference center for adult infectious diseases in the Vilnius district and serves a population of 809,000, which is 27% of the nation’s total population.

Cases of Lyme disease were defined on the basis of documented clinical characteristics, laboratory results, and electrocardiogram and skin biopsy findings. According to clinical presentation, all Lyme disease cases were classified as EM, neuroborreliosis (NB), Lyme arthritis (LA), Lyme carditis (LC), or Lyme dermatitis (LD).

The clinical symptoms were the following: in the erythema migrans group, a typical skin rash; in the Lyme arthritis group, swollen joints and a history of tick bite and/or EM; in the neuroborreliosis group, signs of central nervous system (CNS) involvement and a history of tick bite and/or EM; in the Lyme carditis group, abnormalities (atrioventricular block) in an electrocardiogram (EKG) and a history of tick bite and/or EM. Acrodermatitis was confirmed by skin biopsy. The laboratory criteria were the presence of specific immunoglobulin M (IgM) and immunoglobulin G (IgG) activity in serum detected by immunological tests with enzyme-linked immunosorbent assay (ELISA). (11) Confirmed cases were included for further analysis.

A retrospective cohort study was conducted. Descriptive statistical analysis including frequency tables for categorical data and quantitative data as means (± standard deviation (SD)). Student’s *t*-test and chi-squared test were used to evaluate the differences between two independent quantitative and qualitative datasets, respectively. A two-tailed *p*-value less than 0.05 was considered to be significant. Statistical analysis was performed using R Commander (Rcmdr) version 4.0.0. The study was approved by the Vilnius Regional Biomedical Research Ethics Committee (approval number 158200-17-900-420) 2017 04 11 and the State Data Protection Inspectorate.

## 3. Results

From 2014 to 2016, 1005 patients fulfilled the diagnostic criteria for Lyme disease; 663 patients were women, and 342 patients were men. Most of the patients were middle-aged (51–60 years), with a median age of 53 years. More than a half of the patients exhibited a tick bite (59.6%), mainly on the lower extremities (60.5%) and 96% of the patients had a typical rash, i.e., erythema migrans, which mostly appeared on the lower extremities (67.2%), as single EM (95.8%). In most cases, the tick bite and EM locations were the same (94.7%), Spearman rho = 0.911, *p* < 0.001 (Table 1). About half of the patients (52.5%) were tested for Lyme immunoglobulins in serum, among them, 63.6% were positive for immunoglobulins M and 46.6% were positive for IgG. Clinical symptoms were as follows: arthralgia (33.2%), general weakness (19%), head pain (13.1%), myalgia (11.2%), fever (6.2%), joint swelling (5.9%), head dizziness (5.1%), nervus facialis neuropathy (2.4%), and radiculitis (2.0%). The clinical syndromes of Lyme disease were the following: erythema migrans (94.02%), Lyme arthritis (3.18%), neuroborreliosis (2.28%), Lyme carditis (0.39%), and acrodermatitis (0.09%) (Table 2).

There was no statistical significant difference according to Lyme disease syndromes among different age groups (Table 3).

Most tick bites were observed during “high” season months, from May to September, with the highest incidence in July. Usually, it took more than one week, but less than one month, for erythema migrans to appear.

Concerning erythema migrans, among the patients, predominately, middle-aged women mentioned skin rash (EM). Testing confirmed Lyme immunoglobulins in the serum of 635 (65.7%) women and 332 (34.3%) men (age range 18–91, median age 53.482 (51%)).

The Lyme arthritis group consisted of four (12.5%) men and 28 (87.5%) women (age range 19–80, median age 55). The following joints were affected: knee, 13 (40%); ankle, 9 (28%); hand, 8 (25%); and elbow, 3 (9%). Among the patients, 11 (34%) patients exhibited monoathritis, 19 (59%) patients exhibited oligoarthritis, and in two cases, information about which joint was inflamed was not known. All the patients were positive for Lyme immunoglobulins G.

In the neuroborreliosis group, 16 (69.6%) women and 7 (30, 4%) men (age range 18–77, median age 51) were identified. The main symptoms were the following: nervus facialis neuropathy (47.8%), myalgia (39%), head pain (26%), and radiculitis (21%).

Lyme carditis manifested as an atrioventricular block in three men and one woman, (age range 37–72, median age 51).

Acrodermatitis or Lyme dermatitis was diagnosed in a 61-year-old woman, as a painful, red rash on the hand; the diagnosis was confirmed by histologic investigation.

We were looking for a relationship between the time of erythema migrans appearance after a tick bite and Lyme borreliosis symptoms. A statistically significant relation was observed in the Lyme arthritis group; the longer it took for EM to appear (in our case more than 4 weeks), the higher the probability of developing Lyme arthritis (*p* = 0.013). No statistically significant relationship was observed in the neuroborreliosis group (*p* = 0.260). It was not available to count the relationship in Lyme carditis and Lyme dermatitis groups.

The main drug administered was doxycycline, with a mean treatment duration of 18 days. Amoxicillin was used mostly in cases of pregnancy or elder age. In some cases, one antibiotic was switched with another because of an allergy or inefficacy (Table 4). Regarding which drug was chosen for treating each Lyme syndrome, doxycycline, following by amoxicillin, were most often used for all Lyme disease clinical groups (Table 5).

## 4. Discussion

Our investigation revealed that, in Lithuania, all age groups and both genders can be affected by Lyme disease. Although diagnoses are mostly in middle-aged women, possibly because women are more aware of their health, especially skin changes, men are also at risk for the disease.

Specific Lyme disease syndromes are erythema migrans, facial nerve palsy, radiculoneuritis, carditis, and migratory arthritis [5]. Accompanying symptoms, such as arthralgia, myalgia, headache, and weakness are general and not specific to the disease [6].

In Lithuania, from 2014 to 2016, the average crude Lyme disease incidence rate was 85.4 persons per 100,000 population [4]. The prevalence of Lyme borreliosis (LB) syndromes were the following: EM (94.02%), LA (3.18%), LC (0.39%) LD (0.09%), and NB (2.28%), which was similar to other endemic Europe countries. For example, in France, the mean yearly incidence rate of LB was 53/100,000 (2011–2016), and the LB syndromes were EM (94%), LA (2.5%), and LD (acrodermatitis 0.89% and borrelial lymphocitoma 0.59%) [7]; in Germany, the incidence of Lyme borreliosis ranged from 26/100,000 (2015) to 41/100,000 (2013), with 95% experiencing EM only, 2.7% NB, and 2.1% LA [8].

Lyme disease is a tick-borne disease; however, only 59.6% of patients observe a tick bite, which is easy to miss due to the small size of ticks. Ticks are active during a high season of the year, i.e., May–September, with the peak in July. In our case, 92.5% of patients mentioned a tick bite from May to September and almost one-third of patients reported a tick bite during July. Notably, the tick season in Lithuania is from May to September, but it is much longer in areas further south. Typically, ticks seek hosts in tall grass, and therefore bites are on lower extremities, and in most cases, a tick bite and erythema migrans are at the same site. When a tick bites, a rash (EM) can appear after 2–30 days, usually after 7 days; our investigation revealed that 79.8% of cases of erythema migrans appeared during the first month after a tick bite. When a rash appears within the first few hours or days after tick contact, the rash is a skin reaction to tick salivary agents and does not lead to an infection [9]. It is important to note that, in some cases, the locations of a tick bite and a skin rash may not be the same. Erythema migrans is also a diagnostic marker of the disease. When erythema migrans is seen, no additional laboratory testing is recommended by NICE (National Institute for Health and Care Excellence) guidance [10,11]. In our study, 51% of patients who had EM were tested for Lyme immunoglobulins in serum, which is overdiagnostic.

Lyme arthritis was observed in 3% (*n* = 32) of all cases, mostly as oligoarthritis. Most patients with LA present with monoarticular or oligoarticular inflammatory arthritis affecting one or more large joints, especially the knee [12]. Articular involvement in Lyme disease is characterized by a robust humoral response such that a negative IgG serologic test virtually rules out Lyme arthritis [13]. In our study, all patients were positive for Lyme immunoglobulins G. If adequately treated, the arthritis should last no longer than 3 months [14], and if symptoms stay longer, then another antibiotic course should be administered [15] to relieve symptoms such as pain relief and anti-inflammatory drugs. The use of systemic or intraarticular glucocorticoids is controversial, because they may impair the eradication of the spirochete. If arthritis persists, then, disease modifying antirheumatic drugs can be used including methotrexate, sulfasalazine, and hydroxychloroquine [16].

Neuroborreliosis, a neurological manifestation, occurs in 3–15% of Lyme infections and can manifest as polyradiculitis, meningitis, and (rarely) encephalomyelitis. In our investigation, neuroborreliosis was revealed in 2.2%of all cases, which did not reflect the actual frequency of the syndrome, because it should be diagnosed by cerebrospinal fluid investigation when hospitalized, and not based on a clinical examination during an ambulatory visit. Typical early disease manifestations include painful meningopolyradiculitis of the spinal nerves linked to a unilateral or bilateral facial palsy (Bannwarth’s syndrome), and frequently, radicular pain [17]. Our investigation revealed that almost half of the patients had nervus facialis neuropathy (47.8%); other common symptoms were myalgia (39%), head pain (26%), and radiculitis (21%).

An atrioventricular block (AV) can vary in degree and is the most common presentation of Lyme carditis. LC can also present with acute myocarditis, pericarditis, myopericarditis, endocarditis, and pancarditis. Lyme carditis appears within one to two months (range from 1 to 28 weeks) after the onset of infection. The incidence of cardiac involvement in Lyme disease is estimated to be from 0.3% to 4%, [18]; in our study, the incidence was 0.3%. LC has a strong male predominance of approximately 3:1, in agreement with our finding for Lyme carditis which was observed in three men and one woman. The clinical course of Lyme carditis is generally mild, short term, and in most cases, completely reversible after adequate antibiotic treatment. Other symptoms may be seen, such as faintness, head dizziness, palpitations, and pain in the thorax [19,20]. 

Lyme dermatitis manifests as borrelial lymphocytoma (BL), or acrodermatitis chronica atrophicans (ACA) [21]. The prevalence of Lyme acrodermatitis chronica atrophicans in Europe is about 1–10%, depending on the region; in our study, it was 0.1%. Acrodermatitis chronica atrophicans is a late and long-lasting form of LB, which may be present for many years. It is characterized by red or bluish-red lesions and leads to extensive flaccid atrophy of the skin, which becomes more and more prominent [22]. ACA is most commonly located on the extremities, although it may affect other skin areas, such as the face, and women are diagnosed with ACA two to three times more often than men [23]. We found only one case with diagnosed ACA, which expressed as a painful, red rash on the hand of a 61-year-old woman. It is important to note that acrodermatitis chronica atrophicans can develop from 6 months to 8 years after a tick bite [24].

A diagnosis of Lyme disease can be made based on clinical symptoms and laboratory tests. The main recommendations according to current European case definitions for LB are as follows: Typically, erythema migrans should be diagnosed clinically and does not require laboratory testing; a diagnosis of Lyme neuroborreliosis requires laboratory investigation of the spinal fluid including intrathecal antibody production; and the remaining disease manifestations require testing for serum antibodies to *B. burgdorferi*. Testing individuals with non-specific subjective symptoms is not recommended, because of a low positive predictive value [25,26]. With respect to laboratory testing, an enzyme-linked immunosorbent assay (ELISA) is the first option, and sometimes Western blot (WB) is performed, but only after a positive ELISA test. Positive test results do not always lead to a Lyme disease diagnosis, and negative tests do not definitely rule out a diagnosis; non-infected people may have immunity and test positive, while infected people may have a delay in their antibody response and may test negative [27]. If symptoms of the disease have persisted for one month or longer, there is no need to perform an ELISA for IgM, because of false positive results, which has been called the one-month diagnostic rule for Lyme disease [28]. Antibody levels remain below the detection limits of serologic tests in the first 7 days after exposure; immunoglobulin M (IgM) antibody titers peak between 8 and 14 days after tick contact, but IgM antibodies may never develop in a patient, if appropriate antimicrobial therapy is started early. The IgM antibody response occurs from 1 to 2 weeks, followed by a robust IgG response from 2 to 4 weeks [29]. Because IgM can also cross-react with antigens other than those associated with *B. burgdorferi*, the IgM test is less specific than the IgG test for Lyme disease. The antibodies may persist for months to years, even after successful antibiotic treatment and cure of the disease and, therefore, testing should not be for the purpose of cure control.

Treatment for all Lyme disease patients involves administering antibiotics. Usually, doxycycline is the drug of first choice; treatment durations vary according to the Lyme borreliosis stage, i.e., early localized infection is treated for 10–14 days, and late borreliosis for 21–28 days [30]. In some cases, when symptoms do not resolve after adequate treatment, another antibiotic may be necessary or other symptom relief drugs may be administered.

Regarding prevention, there is no specific immunoprophylaxis against the disease. Since a tick bite can lead to Lyme disease and to other common tick-borne diseases, in Lithuania [31] people can take vaccines only against tick-borne encephalitis. For Lyme disease, only non-specific personal protective measures can be followed such as wearing long-sleeved, brightly colored clothes during outdoor activities, especially in forests, gardens, etc.; using tick repellents on skin or clothing; and carefully checking the body for tick bites, because time matters: the longer a tick sucks, the higher the possibility of infection.

## 5. Conclusions

In Lithuania, the main clinical manifestation of Lyme disease is erythema migrans. Lyme arthritis was seen in 3% of all cases; other syndromes were rare. Erythema migrans was more prevalent among middle-aged women, and the rash appeared mainly on the lower extremities. Lyme arthritis mainly manifested among middle-aged women as an oligoarthritis, and the most affected joint was the knee. Lyme carditis manifested as an atrioventricular block in three men and one woman, and the median age was 51. Acrodermatitis was diagnosed for a 61-year-old woman, as a painful, red rash on the hand. The main drug administered was doxycycline, and the mean treatment duration was 18 days. Finally, when erythema migrans is diagnosed based on clinical symptoms, no additional testing is necessary, followed by antibiotic therapy for 10–14 days. Lyme arthritis, Lyme carditis, and Lyme dermatitis need additional testing and a longer course of antibiotic treatment, i.e., 21–28 days. All tick-borne related neurological symptoms should be treated only after cerebrospinal fluid investigation.

## Figures and Tables

**Table 1:** Frequency of tick bite locations and locations of erythema migrans appearance.

Tick Bite	Erythema Migrans															
	Head		Abdomen		Lower Extremities		Thorax		Genitals		Back		Upper Extremities		Neck	
	*n*	%	*n*	%	*n*	%	*n*	%	*n*	%	*n*	%	*n*	%	*n*	%
Head	5	100.0	0	0.0	0	0.0	0	0.0	0	0.0	0	0.0	0	0.0	0	0.0
Abdomen	0	0.0	41	87.2	4	8.5	1	2.1	0	0.0	0	0.0	0	0.0	1	2.1
Lower extremities	0	0.0	1	0.4	219	97.7	1	0.4	1	0.4	1	0.4	1	0.4	0	0.0
Thorax	0	0.0	1	5.8	2	11.7	11	64.7	0	0.0	2	11.7	1	5.8	0	0.0
Genitals	0	0.0	0	0.0	1	33.3	0	0.0	2	66.6	0	0.0	0	0.0	0	0.0
Back	0	0.0	0	0.0	1	6.25	0	0.0	0	0.0	14	87.5	1	6.25	0	0.0
Upper extremities	0	0.0	0	0.0	4	7.2	0	0.0	0	0.0	0	0.0	51	92.7	0	0.0
Neck	0	0.0	0	0.0	0	0.0	0	0.0	0	0.0	0	0.0	1	33.3	2	66.6

**Table 2:** Descriptive data statistical analysis.

		(*n* = 1005) *n* (%)
Age	Median age (SD)	51.5 (15.86)
	Mediana (min.–max.)	53 (18–91)
Age groups	18–35	187 (18.6)
	36–55	383 (38.2)
	56–70	311 (30.9)
	71–99	124 (12.3)
Gender	Female	663 (66.0)
	Male	342 (34.0)
Observed tick bite		600 (59.6)
Location of tick bite	Head	6 (1.3)
	Neck	3 (0.6)
	Abdomen	56 (12.9)
	Lower extremities	261 (60.5)
	Thorax	18 (4.1)
	Genitals	3 (0.6)
	Back	17 (3.9)
	Upper extremities	67 (15.5)
Erythema migrans (EM)		968 (96.2)
Location of EM	Head	6 (0.7)
	Neck	6 (0.7)
	Abdomen	84 (10)
	Lower extremities	563 (67.2)
	Thorax	34 (4.0)
	Genitals	3 (0.3)
	Back	33 (3.9)
	Upper extremities	108 (12.9)
Location of tick bite and EM	The same	341 (94.7)
	Not the same	19 (5.2)
Single EM		791 (95.8)
Multiple EM		34 (4.1)
Tested for Lyme Immunoglobulins		529 (52.5)
Lyme IgM	Positive	317 (63.6)
	Negative	139 (27.9)
	Marginal	42 (8.4)
Lyme IgG	Positive	168 (46.6)
	Negative	170 (47.2)
	Marginal	22 (6.11)
Clinical disease symptoms	Arthralgia	180 (33.2)
	General weakness	103 (19.0)
	Headache	71 (13.1)
	Myalgia	61 (11.2)
	Fever	34 (6.2)
	Joint swelling	32 (5.9)
	Head dizziness	28 (5.1)
	N. facialis neuropathy	13 (2.4)
	Radiculitis	11 (2.03)
	Nausea	6 (1.1)
	Chill	2 (0.3)
Clinical form of illness	Erythema migrans (EM)	945 (94.02)
	Lyme arthritis (LA)	32 (3.18)
	Neuroborreliosis (NB)	23 (2.28)
	Lyme carditis (LC)	4 (0.39)
	Lyme dermatitis (LD)	1 (0.09)

**Table 3:** Lyme disease syndromes according to age groups.

	EM	NB	LA	LC	LD	Count
18–35	94.1	2.7	3.2	0.0	0.0	187
36–55	93.5	2.3	3.4	0.8	0.0	383
56–70	95.2	1.9	2.6	0.0	0.3	311
71–99	92.7	2.4	4.0	0.8	0.0	124

Pearson’s Chi-squared test X-squared = 7.2419, df = 12, *p*-value = 0.8412.

**Table 4:** Treatment of Lyme disease, treatment duration.

Antibiotic	Frequency	Percent (%)	Treatment Duration—Days Mean (SD)
Amoxicillin	68	6.91	17.8 (10,73)
Amoxicillin, Azitromycin	3	0.30	29.0 (22,63)
Amoxicillin, Doxycicline	10	1.02	32.1 (13,50)
Azitromycin	18	1.83	7.4 (3,14)
Azitromycin, Cefuroxime	1	0.10	16.0 (-) *
Azitromycin, Doxycicline	8	0.81	22.3 (6,22)
Azitromycin, Doxycicline, Ceftriaxone	1	0.10	63.0 (-) *
Ceftriaxone	2	0.20	21.0 (-) *
Ceftriaxone, Doxycicline	2	0.20	23.5 (6,36)
Cefuroxime	8	0.81	14.3 (2,71)
Doxycicline	858	87.19	18.1 (4,69)
Doxycicline, Cefuroxime	2	0.20	29.5 (2,12)
Clarithromycin	3	0.30	8.0 (5,57)

* SD was not counted due to lack of values.

**Table 5:** Antibacterial drugs choice according to Lyme disease clinical syndromes.

	EM	LA	NB	LC	LD	*p*-Value
Doxycicline	833	28	19	2	1	<2.2 × 10^−16^
Amoxicilline	78	2	1	0	0	<2.2 × 10^−16^
Azitromycin	29	1	0	0	0	<2.2 × 10^−16^
Cefuroxime	9	2	0	1	0	1.478 × 10^−5^
Ceftriaxone	0	0	4	1	0	0.01735
Clarithromycine	3	0	0	0	0	0.01735

## Data Availability

The data presented in this study are available on request from the corresponding author. The data are not publicly available due to ethical restrictions.

## References

[1] Steere A.C., Coburn J., Glickstein L., Goodman J.L., Dennis D.T., Sonenshine D.E. (2005). Lyme borreliosis. Tick-Borne Diseases of Humans.

[2] Piesman J., Gern L., Bowman A.S., Nuttall P.A. (2008). Lyme borreliosis in Europe and North America. Ticks: Biology, Disease and Control.

[3] Stanek G., Wormser G.P., Gray J., Strle F. (2012). Lyme borreliosis. Lancet.

[4] Petrulionienė A., Radzišauskienė D., Ambrozaitis A., Čaplinskas S., Paulauskas A., Venalis A. (2020). Epidemiology of Lyme disease in a highly endemic European zone. Medicina.

[5] Dumler J.S. (2001). Molecular diagnosis of Lyme disease: Review and meta-analysis. Mol. Diagn..

[6] Marques A.R. (2010). Lyme disease: A review. Curr. Allergy Asthma Rep..

[7] Septfons A., Goronflot T., Jaulhac B., Roussel V., de Martino S., Guerreiro S., Launay T., Fournier L., De Valk H., Figoni J. (2019). Epidemiology of Lyme borreliosis through two surveillance systems: the national Sentinelles GP network and the national hospital discharge database, France, 2005 to 2016. Euro Surveill..

[8] Enkelmann J., Böhmer M., Fingerle V., Siffczyk C., Werber D., Littmann M., Merbecks S.S., Helmeke C., Schroeder S., Hell S. (2018). Incidence of notified Lyme borreliosis in Germany, 2013–2017. Sci. Rep..

[9] Schriefer M.E. (2015). Lyme disease diagnosis: Serology. Clin. Lab. Med..

[10] Wormser G.P., Dattwyler R.J., Shapiro E.D., Halperin J.J., Steere A.C., Klempner M.S., Krause P.J., Bakken J.S., Strle F., Stanek G. (2006). The clinical assessment, treatment, and prevention of Lyme disease, human granulocytic anaplasmosis, and babesiosis: Clinical practice guidelines by the Infectious Diseases Society of America. Clin. Infect. Dis..

[11] Cruickshank M., O’Flynn N., Faust S.N. (2018). Lyme disease: Summary of NICE guidance. BMJ.

[12] Bockenstedt L.K., Wormser G.P. (2014). Review: Unraveling Lyme disease. Arthritis Rheumatol..

[13] Puius Y.A., Kalish R.A. (2008). Lyme arthritis: Pathogenesis, clinical presentation, and management. Infect. Dis. Clin. N. Am..

[14] Arvikar S.L., Steere A.C. (2015). Diagnosis and treatment of Lyme arthritis. Infect. Dis. Clin..

[15] Puéchal X., Sibilia J. (2009). What should be done in case of persistent symptoms after adequate antibiotic treatment for Lyme disease?. Curr. Probl. Dermatol..

[16] Steere A.C., Angelis S.M. (2006). Therapy for Lyme arthritis: Strategies for the treatment of antibiotic-refractory arthritis. Arthritis Rheum..

[17] Pachner A.R., Steiner I. (2007). Lyme neuroborreliosis: Infection, immunity, and inflammation. Lancet Neurol..

[18] Krause P.J., Bockenstedt L.K. (2013). Cardiology patient pages. Lyme disease and the heart. Circulation.

[19] Kostić T., Momčilović S., Perišić Z.D., Apostolović S.R., Cvetković J., Jovanović A., Barać A., Šalinger-Martinović S., Tasić-Otašević S. (2017). Manifestations of Lyme carditis. Int. J. Cardiol..

[20] Yeung C., Baranchuk A. (2018). Systematic approach to the diagnosis and treatment of Lyme carditis and high-degree atrioventricular block. Healthcare.

[21] Stanek G., Strle F. (2008). Lyme disease—European perspective. Infect. Dis. Clin. N. Am..

[22] Zajkowska J., Czupryna P., Pancewicz S.A., Kondrusik M., Moniuszko A. (2011). Acrodermatitis chronica atrophicans. Lancet Infect. Dis..

[23] Strle F., Wormser G.P., Mead P., Dhaduvai K., Longo M.V., Adenikinju O., Soman S., Tefera Y., Maraspin V., Lotrič-Furlan S. (2013). Gender disparity between cutaneous and non-cutaneous manifestations of Lyme borreliosis. PLoS ONE.

[24] Moniuszko-Malinowska A., Czupryna P., Dunaj J., Pancewicz S., Garkowski A., Kondrusik M., Grygorczuk S., Zajkowska J. (2018). Acrodermatitis chronica atrophicans: Various faces of the late form of Lyme borreliosis. Postepy Dermatologii i Alergologii.

[25] Dessau R.B., van Dam A.P., Fingerle V., Gray J., Hovius J.W., Hunfeld K.-P., Jaulhac B., Kahl O., Kristoferitsch W., Lingren P.-E. (2018). To test or not to test? Laboratory support for the diagnosis of Lyme borreliosis: A position paper of ESGBOR, the ESCMID study group for Lyme borreliosis. Clin. Microbiol. Infect..

[26] Eldin C., Raffetin A., Bouiller K., Hansmann Y., Roblot F., Raoult D. (2019). Parola, P. Review of European and American guidelines for the diagnosis of Lyme borreliosis. Médecine et Maladies Infectieuses.

[27] Mulherin S.A., Miller W.C. (2002). Spectrum bias or spectrum effect? Subgroup variation in diagnostic test evaluation. Ann. Intern. Med..

[28] Branda J.A., Body B.A., Boyle J., Branson B.M., Dattwyler R.J., Fikrig E., Gerald N.J., Gomes-Solecki M., Kintrup M., Ledizet M. (2018). Advances in serodiagnostic testing for Lyme disease are at hand. Clin. Infect. Dis..

[29] Hu L.T. (2016). Lyme disease. Ann. Intern. Med..

[30] Rauer S., Kastenbauer S., Fingerle V., Hunfeld K.-P., Huppertz H.-I., Dersch R. (2018). Lyme neuroborreliosis. Deutsches Ärzteblatt Int..

[31] Radzišauskienė D., Žagminas K., Ašoklienė L., Jasionis A., Mameniškienė R., Ambrozaitis A., Jančorienė L., Jatužis D., Petraitytė I., Mockienė E. (2018). Epidemiological patterns of tick-borne encephalitis in Lithuania and clinical features in adults in the light of the high incidence in recent years: a retrospective study. Eur. J. Neurol..

